# Current Limitations of Electronic Health Record Systems in Supporting Pragmatic Clinical Trials: Insights from the eMERGE Consortium

**DOI:** 10.1101/2025.04.01.25325049

**Published:** 2025-04-03

**Authors:** Kavishwar B. Wagholikar, Jennifer Allen Pacheco, Adam S. Gordon, Atlas Khan, Bahram Namjou Khales, Barbara Benoit, Benjamin J. Kerman, Chunhua Weng, Casey Ta, Cynthia A. Prows, Robert Johnson, Dan M. Roden, David Crosslin, Elizabeth M. McNally, Elizabeth W. Karlson, Frank Mentch, Gail P. Jarvik, Georgia L. Wiesner, Hakon Hakonarson, James J. Cimino, Jeritt G. Thayer, Jordan W. Smoller, Jodell E. Linder, John Connolly, Josh F. Peterson, Josh Cortopassi, Krzysztof Kiryluk, Marwan Hamed, Mary Maradik, Megan J. Puckelwartz, Mohammadreza Naderian, Nephi Walton, Nita Limdi, Devi Priyanka Maripuri, Theresa Walunas, Vivian Gainer, Yuan Luo, Cong Liu, Eimear E. Kenny, Angelica Espinoza, Robb Rowley, Wei-Qi Wei, Shawn N. Murphy

**Affiliations:** Massachusetts General Hospital; Northwestern University Feinberg School of Medicine; Center for Genetic Medicine, Northwestern University; Vagelos College of Physicians & Surgeons, Columbia University; Cincinnati Children’s Hospital Medical Center; Mass General Brigham Inc; Brigham and Women’s Hospital and Harvard Medical School; Columbia University Irving Medical Center; Columbia University; Children’s Hospital Medical Center; The University of Alabama at Birmingham; Vanderbilt University School of Medicine; University of Washington; Northwestern University; Brigham and Women’s Hospital Department of Medicine; The Children’s Hospital of Philadelphia Center for Applied Genomics; University of Washington Medical Center; Vanderbilt University; Perelman School of Medicine of the University of Pennsylvania; University of Alabama-Birmingham; Children’s Hospital of Philadelphia; Massachusetts General Hospital; Vanderbilt University Medical Center; The Children’s Hospital of Philadelphia Center for Applied Genomics; Vanderbilt University Medical Center; University of Alabama at Birmingham Health System Authority; Columbia University; Mayo Foundation for Medical Education and Research; Northwestern University Feinberg School of Medicine; Northwestern University; Mayo Clinic, Rochester, MN; National Institutes of Health; The University of Alabama at Birmingham; Children’s Hospital of Philadelphia; Northwestern University; Mass General Brigham Inc; Northwestern University Feinberg School of Medicine; Boston Children’s Hospital; Icahn School of Medicine at Mount Sinai; Northwestern University Feinberg School of Medicine; National Institutes of Health; Vanderbilt University Medical Center; Mass General Brigham Inc

## Abstract

Pragmatic clinical trials (PCTs) evaluate interventions in real-world settings, often using electronic health records (EHRs) for efficient data collection. We report on the challenges in performing EHR analysis of healthcare provider orders in a PCT within the eMERGE consortium, which investigates the impact of reporting genome-informed risk assessments (GIRA) to over 25,000 patients across 10 academic medical centers. Clinical informaticians conducted a landscape analysis to identify approaches for evaluating the outcomes of GIRA reporting through the EHR. Of 98 identified outcomes, 54 (55.1%) were determined to be difficult to extract because they involved provider orders, which are typically documented in free text or proprietary formats within the EHR and only mapped to standardized codes after the service is completed. These findings highlight a critical barrier in using EHRs to support PCTs. The authors recommend closer collaboration between clinicians and informaticians, improved EHR systems that support standardized order entry, and future use of machine learning to automate analysis of provider behavior in clinical trials.

## Introduction

Pragmatic clinical trials (PCTs) are designed to evaluate the effectiveness of interventions in real-world practice by focusing on diverse populations, flexible protocols, and practical outcomes. [1] Unlike traditional trials in controlled environments, PCTs often leverage electronic health records (EHR) for cost-efficient enrollment and data collection. Here, we report on the challenges in using health-care provider orders in a PCT within the eMERGE consortium, which investigates the impact of reporting genome-based scores. [2] Analyzing provider orders is crucial to distinguish whether gaps in clinical care stem from provider inaction, or other barriers that preclude patients from following up on recommended care.[3]

## Methods

The study includes 25,003 patients recruited from medical practices across 10 academic medical centers. The participants provided electronic consent and age-based assent, completed surveys and submitted biological samples. These data were used to generate a “genome-informed risk assessment” (GIRA) report for 11 common diseases, which was shared with the patients and their healthcare providers. Clinical experts created disease-specific follow-up recommendations in the GIRA report that was returned to high-risk patients. Clinical experts also created a corresponding list of outcome variables for EHR extraction to determine the clinical impact of the GIRA report – whether the proportions of providers recommending risk-reducing interventions and patients undertaking interventions are higher amongst the high-risk patients. Clinical informaticians performed a landscape analysis to identify methods for extracting the outcome variables from the EHR.

## Results

**Figure 1.**
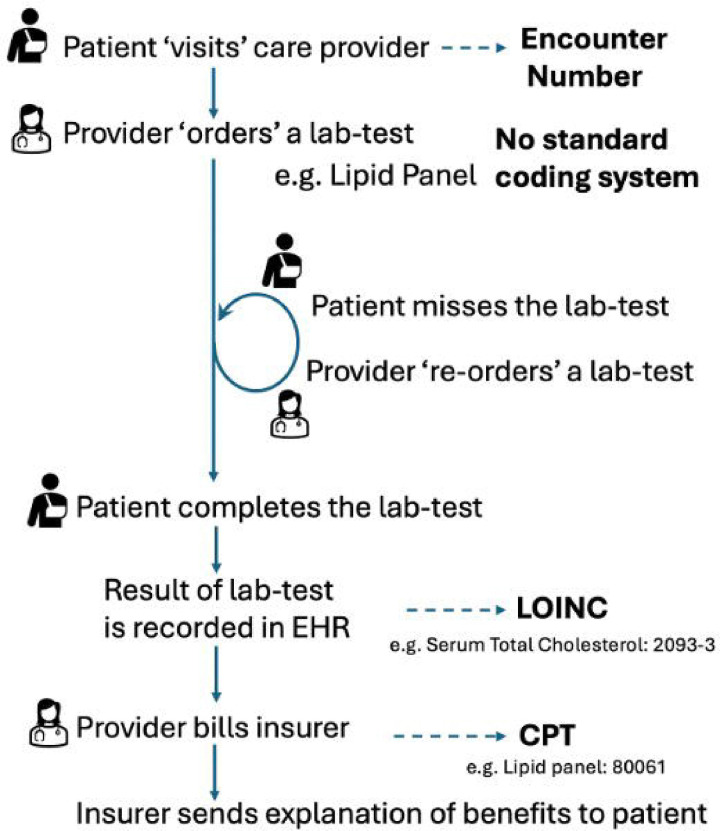
Provider orders for labs and procedures are recorded in the EHR, but they are not coded i.e., mapped to a standard terminology like CPT until after the procedure is completed or the results are reported.

The clinicians identified 98 outcomes for extraction from the EHR. The informaticians considered the use of the Observational Medical Outcomes Partnership (OMOP) Common Data Model (CDM) and terminology mappings for extracting EHR data from each site including demographic, visits, diagnoses, procedures, laboratory-test results and medications.[4] However, the informaticians determined that extracting 54 (55.1%) of the outcomes would be challenging, as they related to provider actions, such as ordering laboratory tests, procedures or referrals (see supplementary file). This difficulty arises because provider orders in EHR systems are recorded in the clinical data model but are not initially coded (see Figure 1).[5] Instead, they are documented using proprietary codes or free text during ordering and are mapped to Current Procedural Terminology (CPT) codes only after the ordered service is completed. Consequently, the group decided to extract the text descriptions associated with the orders and to manually map them to the outcomes.

## Discussion

Our study reveals that EHR systems are not inherently designed to facilitate the analysis of provider ordering, requiring extensive manual effort to determine if the provider adhered to the recommended actions. This finding highlights a major obstacle for pragmatic clinical trials, where it is often essential to decompose the clinical effects at both provider and patient levels– i.e. provider adherence to the study protocol or clinical guideline, and patient adherence to the provider recommendations. Patient behavior can be influenced by various factors, including disagreement with the provider, seeking follow-up care in a different healthcare system, and social determinants of health. Since many orders remain ‘not completed’ due to patient and system-related factors, relying solely on CPT code analysis may underestimate the true clinical impact of the intervention being evaluated in PCTs. To address this issue, we advocate for close collaboration between informaticians and clinicians to decipher the record of provider orders. Additionally, EHR vendors should integrate order entry systems with terminology databases or implement drop-down menus, for recording orders using standardized codes. Until such systems are implemented researchers will need to resort to manual methods to analyze the provider actions. Eventually the use of large language models [6] and machine learning may be useful to monitor and analyze the provider actions.

## Supplementary Material

Supplement 1 **Supplementary file 1.** The clinical outcomes identified by the clinical experts are associated with concepts that need to be extracted from the EHR to assess the outcomes. Among the 98 distinct outcomes, 54 (55.1%) are considered challenging to extract, as they require extracting data on provider actions, such as ordering laboratory tests, procedures, or referrals (as noted in Column F).

## References

[1] Platt R, Bosworth HB, Simon GE. Making Pragmatic Clinical Trials More Pragmatic. JAMA. 2024 Dec 10;332(22):1875–1876. doi: 10.1001/jama.2024.19528. 39356531

[2] Linder JE, Allworth A, Bland HT, Caraballo PJ, Chisholm RL, Clayton EW, Crosslin DR, Dikilitas O, DiVietro A, Esplin ED, Forman S, Freimuth RR, Gordon AS, Green R, Harden MV, Holm IA, Jarvik GP, Karlson EW, Labrecque S, Lennon NJ, Limdi NA, Mittendorf KF, Murphy SN, Orlando L, Prows CA, Rasmussen LV, Rasmussen-Torvik L, Rowley R, Sawicki KT, Schmidlen T, Terek S, Veenstra D, Velez Edwards DR, Absher D, Abul-Husn NS, Alsip J, Bangash H, Beasley M, Below JE, Berner ES, Booth J, Chung WK, Cimino JJ, Connolly J, Davis P, Devine B, Fullerton SM, Guiducci C, Habrat ML, Hain H, Hakonarson H, Harr M, Haverfield E, Hernandez V, Hoell C, Horike-Pyne M, Hripcsak G, Irvin MR, Kachulis C, Karavite D, Kenny EE, Khan A, Kiryluk K, Korf B, Kottyan L, Kullo IJ, Larkin K, Liu C, Malolepsza E, Manolio TA, May T, McNally EM, Mentch F, Miller A, Mooney SD, Murali P, Mutai B, Muthu N, Namjou B, Perez EF, Puckelwartz MJ, Rakhra-Burris T, Roden DM, Rosenthal EA, Saadatagah S, Sabatello M, Schaid DJ, Schultz B, Seabolt L, Shaibi GQ, Sharp RR, Shirts B, Smith ME, Smoller JW, Sterling R, Suckiel SA, Thayer J, Tiwari HK, Trinidad SB, Walunas T, Wei WQ, Wells QS, Weng C, Wiesner GL, Wiley K; eMERGE Consortium; Peterson JF. Returning integrated genomic risk and clinical recommendations: The eMERGE study. Genet Med. 2023 Apr;25(4):100006. doi: 10.1016/j.gim.2023.100006. Epub 2023 Jan 6. . 36621880 PMC10085845

[3] Peh KQE, Kwan YH, Goh H, Ramchandani H, Phang JK, Lim ZY, Loh DHF, Østbye T, Blalock DV, Yoon S, Bosworth HB, Low LL, Thumboo J. An Adaptable Framework for Factors Contributing to Medication Adherence: Results from a Systematic Review of 102 Conceptual Frameworks. J Gen Intern Med. 2021 Sep;36(9):2784–2795. doi: 10.1007/s11606-021-06648-1. Epub 2021 Mar 3. 33660211 PMC8390603

[4] Reich C, Ostropolets A, Ryan P, Rijnbeek P, Schuemie M, Davydov A, Dymshyts D, Hripcsak G. OHDSI Standardized Vocabularies-a large-scale centralized reference ontology for international data harmonization. J Am Med Inform Assoc. 2024 Feb 16;31(3):583–590. doi: 10.1093/jamia/ocad247. 38175665 PMC10873827

[5] Cimino JJ. Putting the “why” in “EHR”: capturing and coding clinical cognition. J Am Med Inform Assoc. 2019 Nov 1;26(11):1379–1384. doi: 10.1093/jamia/ocz125. 31407781 PMC6798564

[6] FHIR-GPT enhances health interoperability with large language models Li Y, Wang H, Yerebakan HZ, Shinagawa Y, Luo Y Nejm Ai 1 (8) 10.1056/aics2300301 PMC12312630 40746832

